# Psychosocial care and experiences in young adults living with early‐onset type 2 diabetes: A narrative review

**DOI:** 10.1111/dme.70352

**Published:** 2026-05-09

**Authors:** Molly Caba, Márcia Carvalho, Angus Forbes, Rita Forde, Kirandip Gill, Mohsin Khalifa, Judith Parsons, Jane Speight, Paula M. Trief, Michelle Hadjiconstantinou

**Affiliations:** ^1^ Diabetes Research Centre, Division of Global, Lifestyle and Metabolic Health, College of Life Sciences University of Leicester Leicester UK; ^2^ Florence Nightingale Faculty of Nursing, Midwifery & Palliative Care King's College London London UK; ^3^ School of Nursing and Midwifery, Brookfield Health Sciences Complex University College Cork Cork Ireland; ^4^ Public Contributor Leciester UK; ^5^ The Australian Centre for Behavioural Research in Diabetes (ACBRD), Diabetes Victoria Carlton Victoria Australia; ^6^ SUNY Upstate Medical University Syracuse New York USA; ^7^ NIHR Leicester Biomedical Research Centre Leicester General Hospital Leicester UK

**Keywords:** psychosocial factors, psychosocial intervention, psychosocial support, review literature, self‐management, type 2 diabetes mellitus, young adult

## Abstract

**Aims:**

To review the literature on psychosocial care and experiences of young adults with early‐onset type 2 diabetes (EOT2D), to identify what is known, current gaps and to develop recommendations to help advance psychosocial care and support for the population.

**Methods:**

We searched Medline (Ovid), Google Scholar and diabetes‐specific journals for English‐language articles focused on psychosocial aspects in young adults (aged 18–45 years) with EOT2D. Two people with lived experience reviewed and commented on the review findings.

**Results:**

Growing evidence indicates that a diagnosis of EOT2D is associated with an increased risk of developing diabetes‐related psychological comorbidities. Experiences of diabetes‐related stigma, compounded by age‐related negative preconceptions, contribute to heightening the psychosocial impact of EOT2D. Some population sub‐groups appear to be more likely to experience adverse psychological effects. However, the evidence base is limited by a dearth of diverse research specifically focused on the psychosocial experiences and needs of this population (e.g., longitudinal and qualitative studies). Adults with EOT2D also experience unmet education, care and support needs relevant to optimising their psychosocial well‐being and diabetes management. Overall, they require enhanced, tailored care and support that is age‐appropriate, person‐centred and responsive to their psychosocial needs. Digital technology and support‐based strategies may help to address current gaps and improve the psychological well‐being of this group, but these require further exploration.

**Conclusions:**

Despite the importance of psychosocial factors in young adults' diabetes management and outcomes, there remain gaps in research and practice and the need for further research, alongside changes in practice.

**What's new?**

**What is already known?**

- Early‐onset type 2 diabetes has significant psychosocial implications for young adults.

**What this study has found?**

- Young adults perceive a lack of tailored psychosocial care and support and report unmet needs.
- Gaps in the evidence remain regarding the psychosocial impact of early‐onset type 2 diabetes.

**What are the implications of the study?**

- Further research on psychosocial aspects of early‐onset type 2 diabetes is needed, alongside more effective, bespoke interventions targeting psychosocial aspects, timely and tailored care and psychosocial support.
- Digital technology and support‐based strategies may help to address current gaps and improve the psychological well‐being of this group.

## 1 INTRODUCTION

Early‐onset type 2 diabetes (EOT2D), defined as a diagnosis of type 2 diabetes (T2D) before the age of 40, is becoming a global health issue.^1^, ^2^ In recent decades, there has been a rapid and significant increase in the incidence and prevalence of EOT2D worldwide, especially in non‐Western populations and regions, including Oceania, South and Central Asia, Latin America and Middle East.^3^ Young adults may now represent 15%–20% of all adults diagnosed with T2D globally.^4^ This is a concerning trend, as compared to later‐onset T2D, EOT2D has a more aggressive phenotype and is associated with a higher risk of early development of diabetes‐related medical complications and mortality.^5^, ^6^ In addition to physical complications, existing evidence suggests that EOT2D is associated with unique psychosocial challenges and an increased risk of mental health comorbidities (the presence of two or more conditions), such as anxiety, depression and diabetes‐related distress.^4^, ^7^ Despite the increase in research on EOT2D in recent years, research on psychosocial aspects of EOT2D remains sparse and fragmented. This is an important gap as diabetes mental health comorbidities can further heighten the risk of premature diabetes‐related complications and mortality, and add to the burden EOT2D places on individuals and health services.^8^ The current limited understanding of psychosocial aspects of EOT2D also hampers any attempts to develop effective, tailored interventions and services for this group. Recent recommendations for the management of EOT2D have highlighted the importance of mental well‐being, identifying it as an essential component of care and a key treatment target.^4^, ^7^ However, given the specific phenotype and unique life circumstances associated with EOT2D, it cannot be assumed that existing psychosocial evidence and care guidelines for T2D will be relevant to this group. Therefore, to address this gap, we present in this paper a narrative review of the literature on psychosocial aspects of EOT2D. This review aims to provide an overview of what is known and what the current gaps are, and identify directions for future research and recommendations for practice.

## 2 METHODS

We conducted a narrative review of the existing literature, as this enables the critical description of existing evidence and derivation of insights to advance the understanding of under‐researched, complex or broad topics. For this review, we included studies that focused on EOT2D (defined as a diagnosis between 18 and 45 years), in line with previous studies.^9^, ^10^, ^11^ Studies only including youth‐onset T2D (<18 years) or late‐onset T2D (>45 years) were excluded. We acknowledge that alternative terminology (e.g., young‐onset or younger‐onset T2D) has also been used to describe young adults diagnosed with T2D <40 years.^5^, ^12^, ^13^ The terminology and criteria used to define the condition are often arbitrary, resulting in differences across the published literature.^14^ By adopting the most flexible definition, we did not exclude studies that have defined EOT2D to the upper age of 45 years. To ensure the comprehensiveness of the review, we conducted searches on Medline (Ovid), Google Scholar and in key diabetes journals using pre‐selected relevant search terms. We searched both databases from inception to June 2025. We also undertook backward and forward citation searches to identify additional relevant studies and considered other studies recommended by co‐authors (see Table 1 for further details). Furthermore, we used a number of systematic procedures to ensure the robustness of the review findings, such as predefined criteria to select studies for inclusion. More specifically, we considered for inclusion peer‐reviewed and non‐peer‐reviewed studies of any design, published in English, and focused on psychosocial aspects of EOT2D. We considered that studies were focused on psychosocial aspects of EOT2D if they examined psychological wellbeing or psychological conditions in people with EOT2D (aged 18–45 years) or diabetes psychological comorbidities experienced by people with EOT2D, personal experiences of diagnosis and living with EOT2D, information/education needs and preferences of people with EOT2D, and social support and social issues affecting diabetes self‐management/mental health and resulting from the diagnosis (e.g., social stigma) of EOT2D. As well as predefined inclusion criteria, two researchers independently screened the titles, abstracts and full texts of all retrieved records and extracted data from selected studies using a piloted form specifically designed for this review. We also worked with two public contributors (KG, MK) living in the United Kingdom, one female and one male, who were diagnosed when they were 24 and 32 years of age, and have been living with EOT2D for 15 and 7 years, respectively. Their involvement aimed to ensure inclusivity by bringing a lived experience perspective into the review findings. We met with the public contributors via MS Teams to discuss the initial draft of the Table S1, followed by remote correspondence for further input by email. The review findings are herein described in line with the Narrative Review Reporting Checklist.^15^

**TABLE 1:** Overview of the search and inclusion and exclusion criteria.

Searches timeframe	Inception to June 2025
Search terms	(‘type 2 diab*’ or T2D* or ‘diabetes type 2*’).ti,ab.
	(‘young* onset’ or ‘early onset’ or youth or ‘youth onset’ or ‘young* adult*’ or ‘emerging adult*’).ti,ab.
	(psychosocial* or ‘psychosocial issue*’ or ‘psychosocial intervention*’ or ‘experience*’ or ‘stigma*’ or ‘diabetes‐related distress’ or ‘distress’ or depression* or ‘eating disorder*’ or ‘disordered eating’ or ‘emotional wellbeing’ or ‘coping’ or ‘quality of life’).mp.
Databases	Medline (Ovid) Google Scholar
Other search methods used	Backward and forward citation searching Targeted electronic searches in key diabetes journals, including Diabetic Medicine, Diabetes Care, The Lancet Diabetes & Endocrinology, Diabetes & Metabolic Syndrome: Clinical Research & Reviews, Diabetes & Metabolic Syndrome: Clinical Research & Reviews, Diabetes Research and Clinical Practice, BMJ Open Diabetes Research & Care, Current Diabetes Reports, Diabetes, Metabolic Syndrome and Obesity, Diabetes Technology & Therapeutics, Journal of Diabetes Research, Diabetes/Metabolism Research and Reviews, Diabetes, Diabetes, Obesity and Metabolism, Cardiovascular Diabetology and Diabetologia Co‐authors recommendations
Inclusion criteria	Articles of any study design published in English with a focus on psychosocial issues among young adults (aged 18–45 years) with early‐onset type 2 diabetes
Exclusion criteria	Articles not published in English Articles without full‐text available or accessible Articles focused on psychosocial aspects in young adults with youth‐onset type 2 diabetes (i.e., diagnosed under the age of 18)

## 3 RESULTS

Our searches led to the identification of 45 relevant studies, most of which were published post 2013, and were conducted in Western, educated, industrialised, rich and democratic (WEIRD) countries (see Table S2 for further details of the reviewed studies). Taken together, these studies indicate that, whilst the psychosocial evidence base for EOT2D is growing, it remains in its infancy in several key areas. Our results summarise the existing evidence regarding the psychosocial impact of EOT2D, and the current psychosocial care and support provided for the population. We also highlight the prevailing gaps in research and practice, and provide recommendations to address these and move the field forward (see Table S1 for a summary).

### 3.1 Psychosocial impact of EOT2D

Overall, current research indicates a high prevalence of emotional and psychological consequences of EOT2D, alongside diabetes‐related stigma, some of which may be shaped by sociodemographic and economic factors.

#### 3.1.1 Emotional and psychological consequences

Existing evidence indicates that the diagnosis of EOT2D can have a significant impact on psychological well‐being and mental health. Mental health comorbidities commonly associated with diabetes, such as depression, diabetes‐related distress and eating disorders, tend to be more prevalent and have a greater severity in young adults with EOT2D than in older adults with T2D,^12^, ^16^, ^17^, ^18^, ^19^, ^20^, ^21^ and the general population.^22^ For example, younger age at diagnosis is associated with an increased risk of depression^16^, ^17^, ^21^, ^23^, ^24^ and diabetes‐related distress^19^, ^20^, ^21^; with one study reporting a 57% increased risk compared to those with later‐onset T2D.^23^ People with EOT2D also frequently report lower levels of self‐compassion and diabetes‐related quality of life. A UK study found that the diagnosis of T2D at a younger age was associated with higher levels of depressive symptoms and diabetes‐specific distress, and lower levels of self‐compassion.^16^ A Korean study found that younger age at diagnosis was independently associated with poorer diabetes‐related quality of life, following adjustment for demographic and medical variables.^24^ EOT2D has also been associated with a potentially increased suicide risk. A nationwide longitudinal cohort study in Japan found an association between diabetes diagnosis and a higher risk of suicide, with the association being larger among those under 40 years of age.^25^ However, this warrants further investigation, as suicide risk assessments were based on self‐reports.

Despite the accumulating evidence underscoring an increased risk of psychosocial issues within the population, evidence gaps remain, particularly regarding the overall prevalence, underlying sources, trajectories and impacts of adverse psychological and mental‐health complications in people with EOT2D.^24^, ^26^ More research is needed not only to improve our understanding of the psychological impact of EOT2D, but also to inform the development of more targeted, evidence‐based management recommendations, models of care and support pathways, interventions and resources.^12^, ^27^, ^28^ To further advance the field, future research should aim to address the limitations of existing research. To date, most of the existing data comes from quantitative cross‐sectional studies.^16^, ^19^, ^20^, ^21^, ^22^, ^24^, ^28^, ^29^, ^30^, ^31^, ^32^, ^33^ Therefore, it is important that future research includes qualitative approaches, longitudinal designs,^19^, ^24^, ^33^, ^34^ and evidence synthesis, to strengthen the evidence base. Future research should equally aim to diversify its focus by exploring a broader range of topics. Most published research tends to focus on certain psychological conditions, such as diabetes‐related distress and depression, with other psychosocial comorbidities, such as eating disorders (i.e., a formal diagnosis according to the diagnostic criteria listed in the Diagnostic and Statistical Manual of Mental Disorders) and disordered eating (i.e., behaviours and psychological distress akin to eating disorders, but which do not meet the specified duration, frequency or intensity for a formal diagnosis),^35^ and diabetes burnout,^36^ remaining largely unexplored. Goldney et al.^18^ suggested that between 10% and 40% of people with T2D may be affected by eating disorders, with those most affected being in early adulthood. They suggest this may in part be due to the psychological impact of living with the condition at a younger age, coupled with the hyper focus on dietary behaviours common in diabetes. However, this suggested association is yet to be explored within research.

In addition, to further our understanding of the mental health impact of EOT2D, efforts should also be made to improve how these impacts are measured. Researchers should conduct further work to develop and validate specific measures for this group, and join efforts to develop a core outcome set (i.e., a standardised minimum set of agreed‐upon outcomes to be measured and reported in all trials of a specific health area)^37^ for research in EOT2D, as it has been done for young adults with type 1 diabetes (T1D).^38^

#### 3.1.2 Diabetes‐related stigma

Current data suggests that the psychological impact of EOT2D may be heightened by anticipated (i.e., expectation or fear of experiencing diabetes stigma), perceived (i.e., belief in, or awareness of, potential diabetes stigma) or experienced (i.e., concrete examples of diabetes stigma) diabetes stigma,^39^ a term that refers to negative social judgement related to having T2D from healthcare professionals, family, friends, peers, co‐workers and the general population. Although we found a paucity of primary research specifically focused on exploring and understanding stigma experienced by the EOT2D population, reports of anticipated, perceived or experienced diabetes stigma in this cohort have been described, particularly in qualitative studies.^12^, ^18^, ^35^, ^40^, ^41^, ^42^, ^43^, ^44^, ^45^, ^46^ Due to the negative connotations surrounding T2D (e.g., T2D is often associated with being ‘old’, ‘fat’, ‘unhealthy’ and ‘lazy’),^41^, ^45^, ^47^ adults with EOT2D frequently described feelings of embarrassment for having diabetes so early in life,^44^, ^48^ shame,^18^, ^35^, ^42^, ^44^ self‐blame,^18^, ^35^, ^44^, ^45^, ^49^ enhanced self‐consciousness,^42^, ^44^ fear of being judged by others^12^, ^35^, ^40^, ^42^, ^43^, ^44^, ^45^, ^46^, ^47^, ^48^, ^50^ and guilt.^42^, ^45^, ^47^ ^,S1^ These feelings and perceptions negatively impacted their acceptance of the diagnosis, which led to feelings of isolation.^S1^ Further, it made them reluctant to share their diagnosis or disclose their condition to others,^12^, ^35^, ^42^, ^45^ which made them feel like a ‘social outcast’,^40^, ^44^ and there was a sense of shame to manage diabetes in public, which compromised their diabetes management.^40^ Hearing negative perceptions and/or remarks from others about diabetes also had a negative impact on their engagement in social activities^44^ (e.g., choosing not to go out or eating out less),^42^ self‐management behaviours^41^ (e.g., pretending not to have dietary restrictions when eating with others),^40^ with healthcare services (e.g., not attending diabetes check‐ups due to feelings of embarrassment)^42^ and with seeking self‐management support^42^, ^43^, ^46^ ^,S1^ (e.g., avoiding diabetes self‐management education due to perceived judgment by others).^43^ These findings are supported by a secondary qualitative analysis looking specifically at experiences of diabetes stigma in this population.^S2^

Although caution is required in the interpretation of existing evidence due to it being obtained from research focused on topics other than stigma, it is evident that experiences from this cohort are amplified and shaped by weight‐ and age‐related assumptions.^39^ Examples include anticipating or experiencing judgment by others, including healthcare professionals, due to misconceptions that T2D is an ‘older person's condition’.^42^, ^43^ This group, who are more likely to live with obesity,^6^ may also experience what is referred to as weight‐related stigma, and compare themselves negatively to others due to their age and weight.^35^, ^48^ ^,S3^ The perceived emphasis of diabetes services being for older people also appears to contribute to feelings of abandonment and stigmatisation by people with EOT2D.^44^ Future research should explore the intersection of diabetes‐related stigma with age‐ and weight‐related stigma, as well as other forms of stigma and discrimination (e.g., gender, race, sexuality, socioeconomic status).^39^ ^,S4,S5^ Future studies should also investigate strategies and/or interventions to address diabetes stigma among this group, as recommended by the Diabetes UK Tackling Diabetes Stigma position statement.^S5^ We found little research focused on initiatives to tackle stigma and its consequences in people with EOT2D. A study in China found that hope, self‐esteem and social support may play a role in reducing the negative impact of stigma on psychological outcomes among young and middle‐aged adults with T2D.^S6^ However, this warrants further investigation, as study participants were all hospitalised and were only followed up for 3 months.

#### 3.1.3 Social and gender inequities influencing psychosocial experiences

The psychosocial impact of EOT2D, and the experiences and needs of people with EOT2D, may also be influenced by sociodemographic and economic factors. Potential differences in the impact of the condition on mental health and wellbeing, as well as the psychosocial experiences and needs across different population subgroups, have been reported across various studies. For instance, multiple studies found women to be at a higher risk than men of experiencing psychosocial issues and emotional problems,^22^, ^30^ including diabetes‐related distress,^30^ depression, anxiety and insomnia.^17^ Although in contrast to these findings, a study conducted in China found that reports of diabetes‐related distress were higher among men with EOT2D.^19^ Bo et al.^22^ further found that perceived stress and depressive symptoms were higher in some subgroups of lower social status and among unemployed people. Similarly, Strandberg et al.^17^ reported that the prevalence of pharmacologically treated depression, anxiety and insomnia was substantially higher for women and those with lower education levels. Research has also revealed potential differences in how effective psychosocial interventions are for different groups of people with EOT2D. The Resilient, Empowered, Active Living with Diabetes (REAL Diabetes) randomised controlled trial^34^ found that adults of low socioeconomic status were less likely to benefit from the intervention than those with higher socioeconomic status, and that women attended fewer intervention sessions, concluding that differential approaches may be needed to better support people from these groups.

Although unsurprising, these findings are of concern as rates of EOT2D are especially higher in these underserved groups, which are already typically more affected by social and health inequalities.^7^ Furthermore, this is likely only one part of the picture, as we found a notable lack of research conducted in low‐ and middle‐income countries, ethnically diverse communities^22^, ^24^, ^26^, ^46^, ^48^, ^49^ and individuals with co‐morbid mental illnesses,^22^ LGBTQIA+ (lesbian, gay, bisexual, transgender, queer/questioning, intersex, asexual, and more) individuals^46^ and pregnant women.^35^ For instance, we identified only one study exploring the experiences of women,^35^ and no studies specifically exploring the experiences of men, or those with diverse gender identities (e.g., transgender, non‐binary). We also observed that younger adults aged 18–25 tend to be underrepresented in the published literature (see Table S1).

Researchers have drawn additional attention to the need for studies specifically focused on the younger end of the 18–45 age range, particularly those aged 18–29 years.^31^ This gap is significant given that the psychosocial needs and experiences of younger adults are likely distinct from those on the older end of the EOT2D age range. For the former, stressors more commonly relate to finances, social life and relationships, whereas for older adults, caring for children or elderly family members and navigating full‐time work may be more prominent stressors.^44^ Overall, more research is needed to better understand psychosocial differences across population subgroups and their specific needs. There should also be a focus on understanding whether the benefits of interventions for this population are equitable across participants of different subgroups and how equitable benefits can be achieved.

### 3.2 Psychosocial care and support

Existing research further indicates that adults with EOT2D have considerable unmet care and support needs, which can negatively impact their psychosocial health and well‐being. There was a strong desire across the studies for more tailored and person‐centred care and support from health services and healthcare teams, particularly concerning education regarding diabetes, its management and mental health.

#### 3.2.1 EOT2D education and care

Current education and models of care appear to contribute to the psychosocial challenges experienced by adults with EOT2D. For example, feelings of frustration,^42^ abandonment, anxiety and stigma^42^, ^44^, ^47^ were reported because of the misalignment of education and care to the population's needs. This is relevant, as adults with EOT2D experiencing psychosocial challenges are more likely to be disengaged from diabetes self‐management behaviours,^19^, ^40^ including physical activity,^41^ healthy dietary behaviours^40^, ^42^ and attending healthcare appointments.^12^, ^18^

Existing evidence indicates that adults with EOT2D desire additional and more age‐appropriate diabetes education beyond the management of the condition, as well as more tailored, person‐centred care.^35^, ^41^, ^42^, ^43^, ^46^, ^47^ ^,S1,S4^ Generally, studies show that adults with EOT2D are not satisfied with the education and information currently provided to them, both pre‐ and post‐diagnosis. Although we found no research on this population specifically focused on diabetes prevention, published studies focused on other topics from the United Kingdom and Australia reported that adults with EOT2D would like further education about diabetes prior to diagnosis to facilitate a focus on reducing modifiable risk factors.^42^ ^,S7^ Increased awareness was seen as important to prevent the development of EOT2D and to help young adults and healthcare professionals detect the condition earlier. Another study conducted in Australia found that young adults with EOT2D reported the need for further awareness and information about T2D and risk reduction amongst the general population and their family/relatives.^42^ Nevertheless, most T2D prevention research and programmes currently available focus on and are more tailored towards older or midlife adults rather than the EOT2D population.^S8^

Current EOT2D education, support and care post‐diagnosis are often perceived to be too generic,^43^ and not sensitive to life stage demands; young adults with T2D often need to juggle multiple commitments, including higher education, full‐time work, family and household responsibilities, burgeoning social lives and romantic relationships.^12^, ^18^, ^40^, ^41^, ^42^, ^44^, ^45^, ^46^, ^48^ ^,S1,S3,S4,S9,S10^ These competing demands often impact time and capacity to engage with health‐promoting behaviours^18^, ^41^, ^42^, ^43^, ^44^, ^45^, ^46^, ^48^ ^,S4,S10^ (e.g., lack of time can foster a reliance on convenience foods low in nutritional value)^44^ and attend diabetes‐related health care appointments.^12^, ^18^ ^,S1,S9^ Healthcare services and support are often perceived as not accommodating of the busy life schedules of young adults,^42^, ^43^ as clinical appointments are often only available within traditional working hours and seldom offered on weekends.^42^

During healthcare appointments, it was also reported that young adults do not feel listened to,^42^, ^44^ or receive empathy or compassion,^35^, ^41^, ^46^ and instead feel patronised, judged or reproached regarding their diabetes management.^42^, ^47^, ^48^ ^,S1^ Diabetes‐related consultations were described as providers just ‘ticking the boxes’ (i.e., covering key points, but not being person‐centred or involving young adults in shared‐decision making).^35^, ^42^ For example, multiple studies highlighted that adults with EOT2D would like further information regarding alternative treatments or non‐pharmacological interventions, but their desire is often met with a lack of support or openness from healthcare providers.^44^, ^46^, ^48^ Young adults also report a dearth of information being provided to them regarding why certain medications are being prescribed, how they work and the potential side effects,^42^ which can result in prescribed medications not being taken.^40^, ^42^, ^43^ This is a particularly important gap given that young adults are likely to be prescribed long‐term medications. Further, young adults feel diabetes services and support, such as diabetes education programmes, are designed with older generations in mind. Topics relevant to young adults tend not to be adequately covered. For instance, fertility and pregnancy were rarely discussed during diabetes appointments,^35^, ^42^, ^43^ unless in relation to starting new diabetes‐related medications.^42^ Goldney et al.^18^ and Sim et al.^43^ further highlight how current EOT2D care and education are not sensitive to the population's cultural needs, especially in relation to dietary information and advice. This is of great importance given the increased risk of EOT2D among individuals of non‐White ethnicity.^4^ ^,S11^

In response to this, enhanced support and adaptations in healthcare delivery are required for this population.^12^, ^30^ There is a need for age‐appropriate information and education and care pathways, and for the provision of holistic, coordinated, patient‐centred care, for example, through bespoke young adult clinical and multidisciplinary care teams.^12^, ^16^, ^18^, ^46^ ^,S1,S12^ To adapt services and improve support, co‐design approaches with people with lived experience is key to make diabetes care and education tailored and inclusive.^12^, ^31^, ^42^, ^43^, ^44^, ^48^ ^,S3,S10^ For example, Celik et al.^35^ reported that women would like to see further discussion of reproductive health (e.g., pregnancy, family planning, contraception, menopause, fertility). Further studies are nonetheless required to better understand the specific needs of certain groups, such as men and individuals of other genders (e.g., LGBTQIA+). To improve attendance at clinical appointments, services should also consider offering greater flexibility,^42^, ^47^ ^,S1^ for example, allowing young adults to book appointments early in the morning or in the evening, or on weekends.^42^ Further, offering virtual appointments^42^ or increasing correspondence through text messages or phone calls may help young adults feel more cared for and supported.^42^ ^,S13^ Research and services for T2D prevention should also consider prioritising early identification and improving prevention support for EOT2D.^S8^ For instance, tailoring education (e.g., diabetes prevention programmes) and information resources provided to those at risk of developing EOT2D, as well as reviewing the appropriateness of existing pathways and guidelines.^42^ In addition, offering enhanced care at certain points, particularly at diagnosis and other critical junctures (e.g., transition to adult services, pregnancy), as well as more regular support,^42^ ^,S3^ may be beneficial for young adults. To address this, the Health Innovation Network UK Report^42^ suggests raising healthcare professionals' and clinical services' awareness of diabetes signs and symptoms to facilitate earlier diagnosis, providing young adults with additional support in the period post‐diagnosis (e.g., longer first and second appointments, 2–3 months apart), and increasing contact time beyond annual reviews.^18^, ^42^ ^,S9^

In light of the existing evidence, increased education, training and support for healthcare and service providers working with adults with EOT2D may also be helpful to address these unmet needs.^35^, ^40^, ^42^, ^46^, ^50^ ^,S1,S7^ Key healthcare provider behaviours desired by young adults, as identified from the literature, include being listened to,^35^, ^42^ ^,S4^ showing empathy,^35^, ^41^, ^43^ using non‐judgemental language,^35^, ^42^, ^43^, ^46^ ^,S1^ providing person‐centred care^35^, ^42^, ^43^, ^46^ ^,S1^ and having an overall positive approach.^42^ ^,S10^ This could be delivered through specific, updated clinical practice guidelines, and the provision of specialised education and training courses for healthcare professionals covering multiple topics relevant to the management of EOT2D.^22^ ^,S10^ The recent Leicester Diabetes Centre's Eden ‘Early Onset Type 2 Diabetes’ training is one example of a specialised course for healthcare providers.^S14^ However, the role of such courses in improving care for EOT2D has not yet been explored. Future research should empirically test the effectiveness of training programmes and initiatives, and further examine healthcare professional‐related factors influencing the provision of care for young adults with EOT2D.^46^

#### 3.2.2 Emotional well‐being support

The psychosocial well‐being of young adults is seldom explored, directly addressed, or supported by healthcare providers during diabetes‐related appointments.^35^, ^42^ In instances where it has been raised, it appears to be in relation to pre‐existing mental health conditions or psychological concerns, rather than in relation to living with EOT2D.^42^ Several findings reported that the clinical focus tends to be on the ‘disease’, with medication,^42^, ^48^ diet, physical activity and weight^42^ taking precedence over psychosocial well‐being and needs, despite the emotional burden of living with T2D from a younger age. This lack of emotional well‐being support often starts at diagnosis, which is a critical juncture as an individual's experience of an EOT2D diagnosis can have a ripple effect regarding how they perceive, manage and accept the condition. Several studies indicate that a diagnosis of EOT2D is often accompanied by feelings of being overwhelmed, alongside shock, confusion, worry and self‐blame,^42^, ^49^ ^,S10^ reactions that can progress to ongoing anxiety, stress and depression.^S4^ Lake et al.^12^ report that high levels of distress at EOT2D diagnosis is associated with increased diabetes‐related distress and depressive symptoms post‐diagnosis. These negative reactions are often driven by a lack of knowledge and understanding regarding the short and long‐term impact of T2D, often related to a lack of information provided by healthcare providers at the time of diagnosis.^42^, ^49^ Gopalan et al.^49^ noted that, compared to other conditions with guidelines for disclosing a diagnosis (e.g., the SPIKES (Setting, Patient's perception, Invitation, Knowledge, Exploring/Empath and Strategy/Summary) model used by practitioners to communicate a diagnosis of cancer),^S15^ there are currently no formal recommended procedures for disclosing an EOT2D diagnosis that aim to provide both support and education to young adults. Future research should explore young adults' experiences of acceptance, adjustment to, and disclosure of the diagnosis of EOT2D, and whether using a formalised model to disclose a diagnosis of EOT2D improves these outcomes.

These findings suggest that the psychological and emotional well‐being aspect of adults with EOT2D should be given greater attention. Greater clinical vigilance and a more proactive approach to detect and address psychological comorbidities, focused on prevention, early detection and intervention, are needed to mitigate negative health consequences.^17^, ^23^, ^28^, ^47^ ^,S12^ This can be especially important for those with a higher risk of poor mental health (e.g., those diagnosed at a younger age^16^, ^17^, ^19^, ^20^, ^24^, ^33^ or with a family history of psychological problems).^S12^ As recommended in guidelines and position statements, regular and ongoing screening for and monitoring of mental health comorbidities should be integrated into routine diabetes care, from early days post‐diagnosis.^12^, ^17^, ^25^, ^26^, ^30^, ^36^ Similarly, psychological support should, where possible, be integrated into the routine care for young adults with EOT2D, and mental health providers be embedded in diabetes care teams and settings.^18^, ^36^, ^42^ ^,S7^ Access to psychiatric services and care should also be facilitated for those in need of a higher level of care.^36^ Regarding treatment, healthcare providers should consider recommending evidence‐based nonpharmacological interventions, such as cognitive behavioural therapy, and medication with less diabetogenic effects, in order to minimise potential adverse metabolic effects of some psychotropic medications,^S12^ as well as explore young adults' preferences regarding medication.

#### 3.2.3 Interventions that address psychosocial needs

Current evidence suggests that existing psychosocial and behavioural interventions do not meet the needs of this group. The number and effectiveness of interventions specifically tailored for this population remains low.^26^, ^36^ ^,S16,S17^ In a review by Lake et al.,^13^ only 2 out of 11 interventions developed for youth and young adults with T2D targeted psychosocial aspects, and only one of these focused on psychosocial well‐being. This intervention developed by Pyatak et al.^34^ for young adults with either T1D or T2D comprised a manualised occupational therapy intervention featuring 12 biweekly sessions, delivered over 6 months, covering living with diabetes, social support, emotions and well‐being, and included goal setting, reminders and problem solving. Its evaluation showed that the intervention significantly improved participants' glycaemic levels (HbA1c), diabetes‐related quality of life and habit strength for blood glucose monitoring. Although the intervention effects did not differ based on diabetes type, there were differences in the glycaemic trajectories of participants, whereby individuals with T2D had a slight deterioration in HbA1c at follow‐up. As those with T1D accounted for a large portion of the sample size, the overall results may not be generalisable to young adults with EOT2D.

In a more recent review by Khavere et al.^S18^ three additional studies were identified that featured a psychosocial component. The first by Dibaiyan et al.^S19^ tested a digital‐based intervention specifically for young adults with T2D. The study delivered online motivational interviewing and diabetes self‐management education sessions through group sessions and WhatsApp. The sessions were delivered by a psychologist, diabetes nurse and nutrition expert, and featured measuring decisional balance, goal setting, problem solving and personal planning (the length, format, content and delivery of the sessions were not explicitly described within the paper). Overall, significant improvements in HbA1c were observed, which was the only outcome of the study. However, given the paucity of information about the study design and intervention content, these results should be interpreted with caution. The second study, by Abdollahi et al.,^S20^ delivered eight 45–60 min Acceptance and Commitment therapy sessions to young adults over 6 months using a standardised protocol (although it is unclear whether this happened in a group or on an individual basis). Improvements in participants' self‐care, self‐efficacy and treatment adherence were observed, although the small sample size (*n* = 30) and self‐reported outcomes should be taken into account when interpreting these results. In the third study, a text message intervention developed by Middleton et al.^S13^ for young adults with T2D delivered a series of information‐based, support‐based and reminder‐based monthly text messages over a 12‐month period. For example, advising on healthy snack options and avenues to receive social support, and providing reminders about upcoming appointments. Overall, clinic attendance increased by over 50%, and there was a significant improvement in self‐efficacy scores in the text message intervention group compared to the standard care control group. However, diabetes‐related distress, stigma and diabetes self‐care did not improve over the duration of the study. As suggested by the authors, to see improvements in these psychosocial factors, a more personalised and concentrated approach is likely needed than can be provided through text messages.

Given the limited number of existing studies and their methodological limitations, there is a need for more effective, evidence‐based, bespoke psychosocial and self‐management interventions for this cohort. Whether developing interventions de novo or adapting existing interventions, it is crucial that interventions are appropriately tailored to this population to ensure their relevance and efficacy, and to promote effectiveness in improving psychosocial well‐being and diabetes‐related outcomes.^13^, ^16^, ^27^, ^30^, ^41^ ^,S7^ Differences in the lived experiences, psychosocial impacts, needs and current levels of support among young adults with EOT2D compared to those with T1D calls for novel, individualised interventions.^S3,S9^ Researchers can draw on existing research to tailor intervention content, method or mode of delivery.^13^, ^27^, ^41^, ^43^ ^,S7^ Khavere et al.^S18^ provide initial evidence on behaviour change techniques that may positively impact health outcomes of young adults, including HbA1c and weight loss. Sim et al.^43^ provided information regarding characteristics of diabetes self‐management education valued by young adults with T2D. In line with behavioural science recommendations, researchers should also develop interventions underpinned by theory, informed by thorough needs assessments, and shaped by people with, or affected by, the condition (e.g., family relatives, carers) and other relevant stakeholders (e.g., diabetes organisations).^13^, ^18^, ^22^

Considering the reported engagement challenges reported in this population,^12^ ^,S1^ novel approaches and methods should be considered and tested in terms of their feasibility. Furthermore, interventions should be systematically evaluated prior to full‐scale implementation, as this is another prevailing gap in the evidence base.^18^, ^43^ Future studies should examine the cost‐effectiveness of novel interventions developed for this population, alongside appropriate clinical and psychosocial outcomes.^18^ Understanding what works, how and why, where, for whom and for how long is crucial to develop more effective interventions and advance research in this group.

#### 3.2.4 Promising avenues to improve psychosocial care and support

We identified two promising avenues for exploration to improve psychosocial care and support for people with EOT2D.

##### Social support

Existing research suggests that social support plays a relevant role in the management of EOT2D, and may be an important target for future interventions for this group.^42^, ^43^, ^45^ ^,S10^ Several studies reported that being open with others about a diagnosis of EOT2D creates opportunities for social support and helps foster acceptance,^45^, ^46^ ^,S4^ alongside coping with the emotional and practical challenges of living with diabetes.^45^ ^,S4^ This in turn promotes psychosocial well‐being and engagement in self‐management behaviours.^45^ ^,S9^ The more individuals are able to talk about their condition and receive support, the more ‘normal’ they feel, and the more they are able to accept T2D as part of their everyday lives.^35^, ^42^, ^43^, ^45^, ^47^ ^,S3,S9^ Receiving support and positive feedback from others also encourages individuals to be more open about their experiences with diabetes.^45^ Talking to others with the condition, particularly peers with similar lived experiences who may be better able to understand and resonate with their unique challenges, has been described as particularly beneficial,^35^, ^42^, ^45^ ^,S4^ and participants across different studies report a desire for enhanced access to peer support. For example, participants in the Health Innovation Network UK report^42^ discussed a desire for additional opportunities for peer support, believing that it could help with understanding the condition, and addressing loneliness and stigma. Participants who did not have or feel able to speak to friends or family described peer or group support as particularly beneficial.^42^ Suggested ways to enhance peer support included having lived experience champions and creating group spaces or forums, such as a local community group or an online community for peer support. Similarly, participants in Savage et al.'s study^S7^ expressed a desire for receiving support from other young people with T2D and suggested that they could support each other through social groups, social outings, regular gatherings combining education and physical activity, email contact and mentoring or buddy systems. Participants further reported a desire for age‐specific education and support groups, as being in groups with older adults made it difficult for young adults to relate to other's experiences.^S7^ Women in Celik et al.'s study^35^ also highlighted the importance of social support, especially peer support from those with similar lived experiences, and reported that speaking with other women made them feel relieved, more confident and less isolated. They further suggested that technology could be leveraged to facilitate access to peer support (e.g., interactive apps). Despite this, we found a lack of support‐based strategies or interventions for this population.

##### Digital technology

Current evidence suggests young adults are using online sources to educate themselves about T2D, perhaps due to a paucity of appropriate information being provided to them by healthcare providers and services. Participants in the Berry‐Price study^S4^ discussed using online social media platforms (e.g., YouTube, Facebook) and medical‐focused websites to learn about the condition and how best to care for it, which helped them feel optimistic and positive about living with the condition long‐term. Similarly, participants in the Sim et al.^43^ study discussed searching the internet for information, although they had concerns about the accuracy of what they read, and shared a preference for individualised information specific to their needs. Healthcare providers in the Armas^S1^ study further discussed how young adults often come to consultations with information obtained through mobile apps (e.g., those that track blood glucose levels) and Google searches, which often promoted positive self‐management behaviours. A study by Jang and Yang^27^ further reported an association between digital health literacy and health‐related quality of life, suggesting that young adults who were able to search for and understand online health information were better able to make informed health decisions. Diabetes organisations could therefore consider developing a curated digital repository with trustworthy information and resources for young adults with EOT2D,^27^, ^42^ given the reports of undertaking their own information seeking through unverified online sources (e.g., social media).

Existing research suggests that there is scope for evidence‐based, provider‐led digital interventions to provide enhanced support. Young adults in the Health Innovation Network UK^42^ report felt there was scope for digital apps to support their health and well‐being and enhance care. Such apps could include educational information about living with diabetes, alongside health trackers, reminders and daily planning functions (e.g., ability to monitor food intake, set alerts to take medication and plan upcoming meals), and methods to book healthcare appointments and communicate with healthcare staff (e.g., to discuss HbA1c results or receive feedback on health information submitted via an app). Participants in the Sim et al.^43^ study also felt that being able to access their appointment history and health results online was helpful for managing their condition, whilst healthcare providers in the Armas^S1^ study advised communicating with young adults through email and text message, where appropriate, could help individuals stay engaged and connected with health services, although this form of communication should not replace in‐person care. Additionally, apps could provide access to an online community of other young adults living with T2D, providing avenues for peer support and helping individuals feel less alone. Participants from the Celik^35^ study further suggested having access to an app that could send supportive and encouraging messages to enhance well‐being and encourage behaviour change, whilst also facilitating peer support through an online chat or forum, which would facilitate understanding and management of the condition. The content to be included in these messages was not explored within the study; however, messages included in the Middleton et al.^S13^ SMS study were found to be supportive and acceptable, providing a foundation for future research in this area. Participants in the Zhao et al.^41^ study further reported that self‐monitoring technologies (e.g., wearable fitness trackers) were an enabler to physical activity, which helped with managing their blood glucose levels.

Overall, existing research indicates that digital technologies to facilitate the delivery of care and support may be acceptable and effective in this population by addressing their reported barriers for managing the condition and accessing and engaging with care (e.g., lack of knowledge, limited contact with HCPs).^41^, ^43^ ^,S1^ Despite this, the number of digitally‐delivered interventions for this population reported in the literature is low.^S18^ The systematic review by Khavere et al.^S18^ identified five interventions for young adults that featured a digital component. The findings of the review are promising as digitally and non‐digitally delivered self‐management interventions for young adults with T2D were found to be equally efficacious. However, these results must be interpreted with caution, given methodological concerns relating to several studies included in the review. Additional research exploring the role of technology to support the management of EOT2D is warranted. Specifically, future research should explore the impact of using technology on psychosocial wellbeing, self‐management behaviours, care provided by healthcare professionals and communication with healthcare staff, as well as how existing and new technologies could be utilised to improve these outcomes. An ongoing longitudinal study is exploring engagement with and impact of a bespoke digital self‐management programme developed specifically for this population, looking at psychosocial outcomes, including diabetes‐related stigma, self‐compassion and self‐efficacy, however no preliminary findings have been published.^S21^

The potential role of interventions that target digital health literacy (to improve young adults' skills and knowledge, their use of online tools and information, and their management of EOT2D), and their impact on wellbeing and self‐management behaviours, also needs further investigation. When studied in underserved groups who reportedly have lower digital health literacy,^27^ future research on digital health interventions should specifically explore the impact of these interventions on health inequities.^27^

## 4 STRENGTHS AND LIMITATIONS

This review has several strengths. First, studies included in the review were identified through a systematic approach following guidelines for narrative summaries. Second, two different authors (MoC and MaC) were involved in the screening and data extraction processes, with regular reflection discussions with an additional author (MH). Third, this review included people with lived experience of EOT2D (KG and MK). The review also has limitations, which must be taken into consideration when interpreting its findings. Studies published in only English were considered for inclusion. Neither the search terms nor the database searches were exhaustive, and evidence was not systematically appraised or weighted for quality, as these were beyond the scope of this review. Furthermore, there is an inherent potential for interpretation bias and selection bias given the nature of the narrative review approach; therefore, details potentially relevant and informative may have been omitted.

## 5 CONCLUSION

Despite the significant growth in research on EOT2D in recent years, psychosocial aspects remain overlooked in research and practice. To the best of our knowledge, this is the first narrative review exclusively dedicated to psychosocial aspects of EOT2D. The current review summarises where we are now and points to where we need to go in the future by outlining important gaps, challenges and opportunities to advance research, clinical practice and policy. With the expected increase in the number of young adults with EOT2D, it is both timely and urgent that researchers, practitioners and policymakers unite efforts and work synergistically to address the gaps described in this review.

## FUNDING INFORMATION

The authors have nothing to report.

## CONFLICT OF INTEREST STATEMENT

None declared.

## Supporting information


Table S1.

Table S2.

Data S1.

## Data Availability

The data that support the findings of this study are available from the corresponding author upon reasonable request.

## References

[1] Diabetes UK . How many people in the UK have diabetes? Accessed March 2, 2026. https://www.diabetes.org.uk/about‐us/about‐the‐charity/our‐strategy/statistics

[2] Wilmot EG , Davies MJ , Yates T , Benhalima K , Lawrence IG , Khunti K . Type 2 diabetes in younger adults: the emerging UK epidemic. Postgrad Med J. 2010;86(1022):711‐718. doi:10.1136/pgmj.2010.100917 20966485

[3] Luk A , Wild SH , Jones S , et al. Early‐onset type 2 diabetes: the next major diabetes transition. Lancet. 2025;405(10497):2313‐2326. doi:10.1016/S0140-6736(25)00830-X 40570862

[4] Savage MJ , Goldney J , Slater T , et al. Management of early‐onset type 2 diabetes in adults: current evidence and future directions. Diabetes Metab J. 2025;49(5):934‐950. doi:10.4093/dmj.2025.0561 40935650 PMC12436035

[5] Lascar N , Brown J , Pattison H , Barnett AH , Bailey CJ , Bellary S . Type 2 diabetes in adolescents and young adults. Lancet Diabetes Endocrinol. 2018;6(1):69‐80. doi:10.1016/S2213-8587(17)30186-9 28847479

[6] Misra S , Ke C , Srinivasan S , et al. Current insights and emerging trends in early‐onset type 2 diabetes. Lancet Diabetes Endocrinol. 2023;11(10):768‐782. doi:10.1016/S2213-8587(23)00225-5 37708901

[7] Misra S , Khunti K , Goyal A , et al. Managing early‐onset type 2 diabetes in the individual and at the population level. Lancet. 2025;405(10497):2341‐2354. doi:10.1016/S0140-6736(25)01067-0 40570864

[8] Ducat L , Philipson LH , Anderson BJ . The mental health comorbidities of diabetes. JAMA. 2014;312(7):691. doi:10.1001/jama.2014.8040 25010529 PMC4439400

[9] Addington KS , Maria K , Nana FH , et al. Incidence of early‐onset type 2 diabetes and sociodemographic predictors of complications: a nationwide registry study. J Diabetes Complicat. 2025;32(2):108942. 10.1016/j.jdiacomp.2024.108942 39700592

[10] Bo A , Thomsen RW , Nielsen JS , et al. Early‐onset type 2 diabetes: age gradient in clinical and behavioural risk factors in 5115 persons with newly diagnosed type 2 diabetes—results from the DD2 study. Diabetes Metab Res Rev. 2017;34(3):e2968. 10.1002/dmrr.2968 29172021

[11] MacLeod K , Gadsby R . Clinical digest 2. Management & prevention of type 2 diabetes. Diabetes Digest. 2004;3(1):23.

[12] Lake AJ , Rees G , Speight J . Clinical and psychosocial factors influencing retinal screening uptake among young adults with type 2 diabetes. Curr Diab Rep. 2018;18(7):41. doi:10.1007/s11892-018-1007-3 29797076

[13] Lake AJ , Bo A , Hadjiconstantinou M . Developing and evaluating behaviour change interventions for people with younger‐onset type 2 diabetes: lessons and recommendations from existing programmes. Curr Diab Rep. 2021;21(12):59. doi:10.1007/s11892-021-01432-1 34902067

[14] Luk AOY , Wu H , Fan Y , Fan B , O CK , Chan JCN . Young‐onset type 2 diabetes—epidemiology, pathophysiology, and management. J Diabetes Investig. 2025;16(7):1157‐1172. doi:10.1111/jdi.70081 PMC12209521 40411309

[15] Green BN , Johnson CD , Adams A . Writing narrative literature reviews for peer‐reviewed journals: secrets of the trade. J Chiropr Med. 2006;5(3):101‐117. doi:10.1016/S0899-3467(07)60142-6 19674681 PMC2647067

[16] Barker MM , Davies MJ , Zaccardi F , et al. Age at diagnosis of type 2 diabetes and depressive symptoms, diabetes‐specific distress, and self‐compassion. Diabetes Care. 2023;46(3):579‐586. doi:10.2337/dc22-1237 36630531 PMC10020022

[17] Strandberg RB , Nilsen RM , Pouwer F , et al. Pharmacologically treated depression, anxiety, and insomnia in individuals with type 2 diabetes: the role of diabetes duration, age, and age at diabetes onset. A Norwegian population‐based registry study from the OMIT cohort. J Psychosom Res. 2025;190:112057. doi:10.1016/j.jpsychores.2025.112057 39955944

[18] Goldney J , Alabraba V , Sarkar P , et al. Designing a regional clinical service for people with early‐onset type 2 diabetes in England. Diabet Med. 2025;42(4):e15479. doi:10.1111/dme.15479 39587392 PMC11929562

[19] Hu Y , Li L , Zhang J . Diabetes distress in young adults with type 2 diabetes: a cross‐sectional survey in China. J Diabetes Res. 2020;2020:4814378. doi:10.1155/2020/4814378 32656266 PMC7321514

[20] Riise HKR , Haugstvedt A , Igland J , et al. Diabetes distress and associated psychosocial factors in type 2 diabetes. A population‐based cross‐sectional study. The HUNT study, Norway. Diabetol Metab Syndr. 2025;17(1):62. doi:10.1186/s13098-025-01631-w 39972441 PMC11837721

[21] Hessler DM , Fisher L , Mullan JT , Glasgow RE , Masharani U . Patient age: a neglected factor when considering disease management in adults with type 2 diabetes. Patient Educ Couns. 2011;85(2):154‐159. doi:10.1016/j.pec.2010.10.030 21112720 PMC3196056

[22] Bo A , Pouwer F , Juul L , Nicolaisen SK , Maindal HT . Prevalence and correlates of diabetes distress, perceived stress and depressive symptoms among adults with early‐onset type 2 diabetes: cross‐sectional survey results from the Danish DD2 study. Diabet Med. 2020;37(10):1679‐1687. doi:10.1111/dme.14087 31335989

[23] Riaz BK , Selim S , Neo M , Karim MN , Zaman MM . Risk of depression among early onset type 2 diabetes mellitus patients. Dubai Diabetes Endocrinol J. 2021;27(2):55‐65. doi:10.1159/000515683

[24] Chung JO , Cho DH , Chung DJ , Chung MY . An assessment of the impact of type 2 diabetes on the quality of life based on age at diabetes diagnosis. Acta Diabetol. 2014;51(6):1065‐1072. doi:10.1007/s00592-014-0677-9 25362507

[25] Komura Y , Inoue K , Ishimura N , et al. Diabetes and suicide: a nationwide longitudinal cohort study among the Japanese working‐age population. J Epidemiol Community Health. 2025;79(5):340‐346. doi:10.1136/jech-2024-222701 39603686

[26] Dibato J , Montvida O , Ling J , Koye D , Polonsky WH , Paul SK . Temporal trends in the prevalence and incidence of depression and the interplay of comorbidities in patients with young‐ and usual‐onset type 2 diabetes from the USA and the UK. Diabetologia. 2022;65(12):2066‐2077. doi:10.1007/s00125-022-05764-9 36059021 PMC9630215

[27] Jang Y , Yang Y . Effects of e‐health literacy on health‐related quality of life in young adults with type 2 diabetes: parallel mediation of diabetes self‐efficacy and self‐care behaviors. Appl Nurs Res. 2025;82:151917. doi:10.1016/j.apnr.2025.151917 40086937

[28] Park JI , Baek H , Kim SW , et al. Questionnaire‐based survey of demographic and clinical characteristics, health behaviors, and mental health of young Korean adults with early‐onset diabetes. J Korean Med Sci. 2021;36(26). doi:10.3346/jkms.2021.36.e182 PMC8258240 34227263

[29] Berge LI , Riise T , Fasmer OB , Lund A , Oedegaard KJ , Hundal O . Risk of depression in diabetes is highest for young persons using oral anti‐diabetic agents. Diabet Med. 2012;29(4):509‐514. doi:10.1111/j.1464-5491.2011.03530.x 22133020

[30] Browne JL , Nefs G , Pouwer F , Speight J . Depression, anxiety and self‐care behaviours of young adults with type 2 diabetes: results from the International Diabetes Management and Impact for Long‐term Empowerment and Success (MILES) study. Diabet Med. 2015;32(1):133‐140. doi:10.1111/dme.12566 25131861

[31] Browne JL , Scibilia R , Speight J . The needs, concerns, and characteristics of younger Australian adults with type 2 diabetes. Diabet Med. 2013;30(5):620‐626. doi:10.1111/dme.12078 23181664

[32] Bo A , Jensen NH , Bro F , Nicolaisen SK , Maindal HT . Higher patient assessed quality of chronic care is associated with lower diabetes distress among adults with early‐onset type 2 diabetes: cross‐sectional survey results from the Danish DD2‐study. Prim Care Diabetes. 2020;14(5):522‐528. doi:10.1016/j.pcd.2020.02.003 32169500

[33] Cha E , Son NH , Joung KH , et al. Differences in patient‐reported and clinical characteristics by age group in adults with type 2 diabetes. Worldviews Evid‐Based Nurs. 2024;21(4):467‐476. doi:10.1111/wvn.12715 38500018

[34] Pyatak EA , Carandang K , Vigen CLP , et al. Occupational therapy intervention improves glycemic control and quality of life among young adults with diabetes: the resilient, empowered, active living with diabetes (REAL diabetes) randomized controlled trial. Diabetes Care. 2018;41(4):696‐704. doi:10.2337/dc17-1634 29351961 PMC5860833

[35] Celik A , Sturt J , Temple A , Forbes A , Forde R . ‘No one ever asks about something that actually is relevant to my life’: a qualitative study of diabetes and diabetes care experiences of young women with type 2 diabetes during their reproductive years. Diabet Med. 2023;40(3):e15017. doi:10.1111/dme.15017 36448267 PMC10107676

[36] de Wit M , Gajewska KA , Goethals ER , et al. ISPAD clinical practice consensus guidelines 2022: psychological care of children, adolescents and young adults with diabetes. Pediatr Diabetes. 2022;23(8):1373‐1389. doi:10.1111/pedi.13428 36464988 PMC10107478

[37] Williamson PR , Altman DG , Blazeby JM , et al. Developing core outcome sets for clinical trials: issues to consider. Trials. 2012;13(1):132. doi:10.1186/1745-6215-13-132 22867278 PMC3472231

[38] Byrne M , O'Connell A , Egan AM , et al. A core outcomes set for clinical trials of interventions for young adults with type 1 diabetes: an international, multi‐perspective Delphi consensus study. Trials. 2017;18(1):602. doi:10.1186/s13063-017-2364-y 29258565 PMC5735534

[39] Speight J , Holmes‐Truscott E , Garza M , et al. Bringing an end to diabetes stigma and discrimination: an international consensus statement on evidence and recommendations. Lancet Diabetes Endocrinol. 2024;12(1):61‐82. doi:10.1016/S2213-8587(23)00347-9 38128969

[40] Wong SKW , Soon W , Griva K , Smith HE . Identifying barriers and facilitators to self care in young adults with type 2 diabetes. Diabet Med. 2024;41(4):e15229. doi:10.1111/dme.15229 37767739

[41] Zhao X , Duaso M , Ghazaleh HA , Guo X , Forbes A . Barriers and facilitators to physical activity in people with young‐onset (aged 18‐40 years) type 2 diabetes: a qualitative study. J Clin Nurs. 2025;34(6):2386‐2399. doi:10.1111/jocn.17691 39963930 PMC12125533

[42] Health Innovation Network South London . Early onset type 2 diabetes: lived experience insights report. 2024. Accessed March 2, 2026. https://healthinnovationnetwork.com/wp‐content/uploads/2025/02/EOT2D‐patient‐insights‐report‐v8.pdf

[43] Sim RRJ , Soon W , Smith HE , Griva K , Wong SKW . Understanding the preferences of young adults with type 2 diabetes mellitus with regard to diabetes self‐management education: a qualitative study. BMJ Open. 2024;14(7):e086133. doi:10.1136/bmjopen-2024-086133 PMC11227779 38964801

[44] Croke S , Volkmann AM , Perry C , et al. What are the perspectives of adults aged 18–40 living with type 2 diabetes in urban settings towards barriers and opportunities for better health and well‐being: a mixed‐methods study. BMJ Open. 2023;13(9):e068765. doi:10.1136/bmjopen-2022-068765 PMC10514606 37730399

[45] Nielsen MH , Jensen AL , Bo A , Maindal HT . To tell or not to tell: disclosure and self‐management among adults with early‐onset type 2 diabetes: a qualitative study. Open Diabetes J. 2020;10(1):11‐19. doi:10.2174/1876524602010010011

[46] Chauhan R , Davies MJ , May C , et al. Using normalisation process theory to understand implementation of effective early‐onset type 2 diabetes treatment and care within England: a qualitative study. BMC Health Serv Res. 2025;25(1):422. doi:10.1186/s12913-025-12616-w 40128789 PMC11931738

[47] Rasmussen B , Terkildsen Maindal H , Livingston P , Dunning T , Lorentzen V . Psychosocial factors impacting on life transitions among young adults with type 2 diabetes: an Australian—Danish qualitative study. Scand J Caring Sci. 2016;30(2):320‐329. doi:10.1111/scs.12248 26037014

[48] Health Innovation Manchester . Cities changing diabetes: Mapping the challenge of type 2 diabetes in greater Manchester. 2022. Accessed March 2, 2026. https://healthinnovationmanchester.com/wp‐content/uploads/2022/06/Cities‐Changing‐Diabetes‐Manchester‐report‐final‐June‐2022.pdf

[49] Gopalan A , Blatchins MA , Altschuler A , Mishra P , Fakhouri I , Grant RW . Disclosure of new type 2 diabetes diagnoses to younger adults: a qualitative study. J Gen Intern Med. 2021;36(6):1622‐1628. doi:10.1007/s11606-020-06481-y 33501523 PMC7837080

[50] Wong SKW , Soon W , Griva K , Smith HE . Diabetes knowledge, self‐efficacy and dietary, psychological and physical health barriers: comparing young and usual‐onset type 2 diabetes. Diabet Med. 2024;41(3):e15207. doi:10.1111/dme.15207 37597247

