# Supplementary material for: Psychosocial care and experiences in young adults living with early‐onset type 2 diabetes: A narrative review
Source: Diabet Med. 2026 May 9;43(7):e70352. doi: 10.1111/dme.70352 (PMC13257897; doi:10.1111/dme.70352)
Supplement: Supplementary file 1 — Table S1. [file DME-43-e70352-s001.docx]

**Supplementary Table 1: Summary of the Literature (Gaps and Recommendations) accompanied by public and patient involvement** **(PPI) contributors’ perspectives**

The literature summary and the PPI contributors’ perspectives are presented side-by-side to show their alignment with the findings of the review, and to highlight PPI member’s unique contributions and views about the review’s key findings also.^1^

| **Literature** | **PPI contributors’ perspectives on the literature** |
| --- | --- |
| **Gaps and Recommendations** | |
| ***3.1. Psychosocial impact of EOT2D***  ***3.1.1. Limited evidence on emotional and psychosocial consequences***  ***Gaps***   - Limited number of studies published on this topic (e.g., diabetes-related distress, depression, disordered eating, stigma). - Limited understanding of how psychosocial impact may differ across 18-45 age range, particularly in the 18-30 years age group. - Limited research exploring the lived experiences of young adults (e.g., qualitative studies).   ***Recommendations***   - More global studies, particularly focusing on psychosocial and behavioural aspects of EOT2D. - Additional research on methods for identifying and monitoring psychosocial wellbeing, and providing psychosocial support.   ***3.1.2. Diabetes-related stigma***  ***Gaps***   - Lack of primary research on stigma experienced by young adults. - Lack of evidence-based, effective strategies to tackle stigma. - Lack of research exploring the role of peer support for combating diabetes stigma. - Limited understanding of the psychosocial and behavioural impact of stigmatising healthcare professional interactions (e.g., judgmental language).   ***Recommendations***   - Further explore diabetes- and weight related stigma; and discrimination experienced by young adults. - Identify and test strategies to combat stigma.   ***3.1.3. Social and gender inequities influencing psychosocial experiences***  ***Gaps***   - Limited understanding of psychosocial challenges across population subgroups (e.g., gender, socioeconomic, ethnic and cultural groups).   ***Recommendations***   - More research focused on under-represented groups, including ethnic minority groups and groups from low- and middle-income countries.   ***3.2. Psychosocial care and support***  ***3.2.1. EOT2D education and care***  ***Gaps***   - Existing care is not tailored to the needs faced by young adults. - Topics relevant for young adults (e.g., reproductive health) are not addressed within diabetes care.   ***Recommendations***   - Improve early identification of those at risk of developing EOT2D. - Tailor education and information resources about EOT2D. - Provide age-appropriate care, holistic, coordinated care, particularly at diagnosis and other transition moments. - Introduce support from healthcare providers between appointments and offer flexibility (e.g., appointments outside of office hours). - Invest in building positive, respectful relationships with young adults. - Provide specialised education and training on EOT2D, such as: reproductive health, diabetes stigma, emotional aspects of EOT2D.   ***3.2.2. Emotional well-being support***  ***Gaps***   - Healthcare focused on the ‘disease’, rather than the person, often overlooking psychosocial challenges. - Lack of formalised methods for disclosing EOT2D diagnosis to help ensure young adults feel cared for and supported.   ***Recommendations***   - Investigate barriers and enablers faced by healthcare providers to provide psychosocial care to young adults. - Restructure healthcare systems with a more proactive approach to identify psychological problems, focused on prevention, early detection and treatment, particularly amongst those at higher risk. - Integrate screening of mental health into routine EOT2D care. - Make psychological support part of routine EOT2D care and improve access to mental health services (e.g., cognitive behavioural therapy). - Include mental health professionals in diabetes care teams.   ***3.2.3. Interventions that address psychosocial needs***  ***Gaps***   - Limited number of programmes specifically designed for this group.   ***Recommendations***   - Develop effective, tailored psychosocial and self-management interventions underpinned by theory and needs assessments, and informed by those with lived experience. - Conduct research on how to engage young adults in research and care. - Intervention evaluation studies (e.g., clinical and cost-effectiveness, and acceptability).   ***3.2.4. Promising avenues to improve psychosocial care and support***  ***3.2.4.1. Social support***   - Conduct research exploring the effectiveness of peer support strategies, including local and online community groups.   ***3.2.4.2. Digital technology***   - Conduct additional research exploring the role of technology to support the management of diabetes, and improve communication with healthcare professionals and care. - Further research exploring digital interventions among underserved groups. - Introduce digital libraries with trustable information and resources. | - The unrecognised importance of psychosocial issues:   - Psychosocial issues are prominent for young adults with EOT2D, yet there is limited evidence to support this.   - Socialising is key in early adulthood, but hard to navigate due to feelings of shame related to disclosing a diagnosis.   - Young adults may be in denial and dismiss the diagnosis, which can lead to disengagement with care and self-management. - Need for additional studies:   - Mental health issues in diabetes is still in its infancy.   - Explore the different ways diabetes management might influence mental health (e.g., impact on travelling and driving).   - A better understanding on mental health screening and prevention. - The image of diabetes and its implications for young adults with EOT2D:   - Young adults commonly experience diabetes-related stigma, both internalised and social stigma, with stigma described as being ‘everywhere’.   - Stigma can lead to concealing the condition from others, as it is still seen as an ‘older person disease’.   - Some minority groups, such as South Asian, might be more affected by stigma.   - Social media and public discourse contribute to diabetes stigma. - Importance of communication campaigns:   - Young adults believe that communication campaigns are important to debunk and combat myths about diabetes causes, management and treatments, and change the public narrative about the condition.   - Language recommendations for the media to promote more positive representations of people with diabetes of all shapes and ages.   - Educate children and young people about long-term health conditions in schools to raise awareness and help foster empathy. - Gender differences and the unacknowledged psychosocial impact of EOT2D in men:   - Men may feel the pressure to hide their diagnosis, especially those from ethnic minority background.   - Men face difficulties to share psychosocial challenges of living with T2D.   - Men’s health and sexual health is rarely addressed. - Greater focus to be placed on women’s health topics, such as gestational diabetes, endometriosis and polycystic ovary syndrome. - Explore psychosocial needs in ethnic/religious groups, and across countries, and other groups (i.e., neurodiversity). - Lack of tailored approach to care:   - Current care tends to be tailored to older adults, leading to strong unmet educational, psychosocial and support needs for young adults.   - Healthcare often uses scare-focused communication strategies, to encourage engagement with care. This may lead to higher levels of anxiety, denial and rebellious behaviour. - Importance of tailored, holistic care:   - Care provided should be holistic alongside other chronic conditions.   - A more integrated approach, with a better articulation between clinical and community-based services facilitated by social prescribers.   - Additional support to deal with diagnosis as they are simultaneously undergoing multiple life transitions (e.g., aftercare appointment a few weeks after diagnosis).   - Increase the availability and flexibility of appointments (e.g., offering after-hours appointments).   - Improve education about diabetes, its associated risks and self-management (including self-monitoring of blood glucose levels). - Importance of additional training and education for healthcare providers:   - Young adults believe it is important to educate and train healthcare providers on EOT2D, including pharmacists. - Importance of psychosocial support and specialised psychological care:   - Address psychological factors which are key to diabetes self-management (i.e., dietary and physical activity behaviours).   - Psychosocial issues and psychological health are often overlooked, with current care being mainly focused on physical health.   - Psychosocial aspects should be discussed and addressed at the time of diagnosis and throughout the course of diabetes.   - Access to specialised psychological care is a must.   - Mental health support should be a standard part of EOT2D care to help support diabetes management. - The prevalence of a ‘one-size-fits-all’ approach and lack of tailored programmes:   - Current self-management education and support programmes do not meet the specific needs of young adults. This is because programmes often take place during business hours, requiring young adults to take time off work to attend. - Desire for specific, bespoke interventions:   - There is limited time with general practitioners to cover EOT2D self-management, such as diet, exercise.   - Group-based interventions may limit attendance due to stigma, despite being beneficial to share experiences with others. - Peer support groups, forums or helplines may be helpful. - Potential benefits of digital technology:   - Digital technologies could be beneficial to improve participation and engagement with care and support, particularly for those who feel stigmatised and are managing multiple competing life demands.   - Mental health support online and continuous glucose monitoring can be informative and helpful, and should be free to those with EOT2D.   - Readily available digital app with information about diet and exercise tailored to people with EOT2D would help support self-management behaviours. |

PPI can be defined as research conducted with or by patients and/or members of the public, instead of conducted on, for, or about them.

**References**

1. Bailey K, Allemang B, Vandermorris A, et al. Benefits, barriers and recommendations for youth engagement in health research: combining evidence-based and youth perspectives. *Research Involvement and Engagement*. 2024-09-02 2024;10(1)doi:10.1186/s40900-024-00607-w
