# Supplementary material for: Psychosocial care and experiences in young adults living with early‐onset type 2 diabetes: A narrative review
Source: Diabet Med. 2026 May 9;43(7):e70352. doi: 10.1111/dme.70352 (PMC13257897; doi:10.1111/dme.70352)
Supplement: Supplementary file 2 — Table S2. [file DME-43-e70352-s003.docx]

**Supplementary Table 2: Characteristics of the studies reviewed** **(*N =* 45)**

| **Author, year** | **Title** | **Country** | ***n* people with early-onset type 2 diabetes (% total sample)** | **Sample characteristics** | **Aim(s)** | **Study type (design)** |
| --- | --- | --- | --- | --- | --- | --- |
| Diabetes Australia, 2006 | Young adults with diabetes needs analysis | Australia | *n* = 49 (12%) | Mean age: 28.8 years  Age at diagnosis:  25+ years: 54%  20-25 years: 26%  16-20 years: 16%  11-15 years: 2%  Female: 67%  Aboriginal and Torres Strait Islander: 14% | To identify young adults (16-35 years) with diabetes’ concerns and emerging issues | Mixed-methods |
| Savage et al., 2009 | The education and information needs of young adults with type 2  diabetes: A qualitative study | Australia | *n* = 13 (100%) | Age range: 26-44 years  Mean age: 38 years (SD = 5.9)  Mean diabetes duration: 29 months (SD = 32.3) | To identify the education and information needs and preferred information delivery mode of people with type 2 diabetes aged 25 to 45 to enable them to effectively self-manage their diabetes | Qualitative (Cross-sectional) |
| Hessler et al., 2011 | Patient age: A neglected factor when considering disease management in adults with type 2 diabetes | United States of America | Not reported | Mean age: 57.80 years (SD = 9.85)  Female: 56.9%  Mean diabetes duration: 8.14 years (SD = 7.50)  Race/ethnicity:  Asian American: 16.8%  African American: 20.5%  Hispanic: 19.3%  Non-Hispanic white: 36.7%  Other: 6.7% | To examine age differences in key aspects of diabetes management, controlling for potentially confounding patient characteristic (behavioural self-management, stress, depressed affect, and self-efficacy) and HbA1c | Quantitative (Cross-sectional) |
| Berge et al., 2012 | Risk of depression in diabetes is highest for young persons using oral anti-diabetic agents | Norway | Not reported | *N* = 3 434 233  Men: 49.03% | To investigate the risk of depression in different types of treatment for diabetes and in subgroups of age and sex | Quantitative (Cross-sectional) |
| Browne et al., 2013 | The needs, concerns, and characteristics of younger  Australian adults with type 2 diabetes | Australia | *n* = 149 (100%) | Age range: 18-39 years  Mean age: 32.33 years (SD = 4.83)  Women: 61.8%  Diabetes duration  range: 0-15 years  Mean diabetes duration: 2.86 years (SD = 3.36 years)  Aboriginal or Torres Strait Islander origin: 6% | To understand the emotional well-being and unmet needs of younger adults with type 2 diabetes, and to inform service provision for this group | Qualitative (Cross-sectional) |
| Browne et al., 2014 | Depression, anxiety and self-care behaviours of young adults with type 2 diabetes: Results from the International Diabetes Management and Impact for Long-term Empowerment and Success (MILES) Study | Australia and the Netherlands | *n =* 93 | Mean age: 33 (SD = 6)  Women: 72%  Mean diabetes duration: 5 years (SD = 5) | To compare the emotional well-being and self-care behaviours of young adults with type 2 diabetes with those of older adults with type 2 diabetes and young adults with type 1 diabetes | Quantitative (Cross-sectional) |
| Chung et al., 2014 | An assessment of the impact of type 2 diabetes on the quality of life based on age at diabetes diagnosis | South Korea | *n* = 116 (49.2%) | Mean age: 46.3 years  Men: 56.9%  Diabetes duration: 63.8% ≥ 5 years | To investigate differences in the perceived impact of diabetes on  quality of life between Korean adults diagnosed with type 2 diabetes before the age of 40 and diagnosed at an older age | Quantitative (Cross-sectional) |
| Rasmussen et al., 2016 | Psychosocial factors impacting on life transitions among  young adults with type 2 diabetes: An Australian – Danish  qualitative study | Australia and Denmark | *n* = 26 (100%) | Age range: 19-42 years  Median age: 34 years  Women: 50%  Diabetes duration range: 0.5-19 years  Diabetes duration median: 5.6 years | To explore similarities and differences in impact of psychosocial factors on Australian and Danish young adults with type 2 diabetes, and to identify unmet support needs during life transitions | Qualitative (Cross-sectional) |
| Armas, 2017 | Primary care providers observations on diabetes management for young adults in Ontario: Barriers and enablers to care and patient-provider communication | Canada | *n* = 2 (15.38%) | Both participants were female in their 30s. One participant had lived with diabetes for 9 years and the other for 3 years | To explore barriers and facilitators to self-management care and communication between young adults with type 2 diabetes and their primary care  providers | Mixed-methods |
| Lake et al., 2018a | Developing and evaluating behaviour change interventions for people with younger-onset type 2 diabetes: Lessons and recommendations from existing programmes | N/A | N/A | N/A | To synthesise lessons learned and recommendations from published evaluations of younger-onset focused health behaviour change interventions, and to identify challenges and to propose solutions using case studies from  own experience | Rapid literature review |
| Lake et al., 2018b | Clinical and psychosocial factors influencing retinal screening uptake  among young adults with type 2 diabetes | N/A | N/A | N/A | To summarise the clinical and psychosocial barriers influencing young adults with type 2 diabetes (18-39 years) uptake of diabetic retinopathy screening | Literature review |
| Pyatak et al., 2018 | Occupational therapy intervention improves glycemic control and quality of life among young adults with diabetes: the Resilient, Empowered, Active Living with Diabetes (REAL Diabetes) randomised controlled trial | United States of America | *n =* 20 (25%) | Total sample  Mean age: 22.6 years (SD = 3.5)  Female: 63%  Mean diabetes duration: 9.7 years (SD = 5.8)  Ethnicity  White: 10%  Black: 10%  Hispanic/Latino:78%  Other: 2% | To assess the efficacy of a manualized occupational therapy intervention (Resilient, Empowered, Active Living with Diabetes (REAL Diabetes) to improve glycaemic control and psychosocial well-being among ethnically diverse young adults with low socioeconomic status with type 1 or type 2 diabetes | Quantitative (Randomised controlled trial) |
| Komura et al., 2019 | Diabetes and suicide: A nationwide longitudinal  cohort study among the Japanese working age population | Japan | Not reported | *n* with diabetes: 2 105 136  Total sample  Mean age: 52 years (SD = 11.9)  Men: 66.9% | To investigate the association between diabetes and  suicide in Japan | Quantitative (Longitudinal cohort study) |
| Bo et al., 2019 | Prevalence and correlates of diabetes distress, perceived stress and depressive symptoms among adults with early-onset type 2 diabetes: Cross-sectional survey results from the Danish DD2 study | Denmark | *n =* 216 (100%) | Age range: 38-44 years Median age: 42  Female: 48%  Diabetes duration range: 3-7 years  Mean diabetes duration: 5 years | To determine the prevalence of diabetes distress, perceived stress and depressive symptoms and to investigate their associations with sociodemographic and clinical characteristics | Quantitative (Cross-sectional) |
| Wong et al., 2019 | Effectiveness of self-management interventions in young adults with type 1 and 2 diabetes: A systematic review and meta-analysis | N/A | N/A | N/A | To assess the effectiveness of self-management interventions for young adults (aged 15–39 years) with type 1 or type 2 diabetes | Systematic review and meta-analysis |
| Bo et al., 2020 | Higher patient assessed quality of chronic care is associated with lower diabetes distress among adults with early-onset type 2 diabetes: Cross-sectional survey results from the Danish DD2-study | Denmark | *n =* 197 (100%) | Mean age: 42.1 years (IQR = 38.2-44.4)  Men: 51.8%  Mean diabetes duration: 5.2 years (IQR = 3.4-6.5)  Foreign born: 5.6% | To investigate the perceived quality of chronic care, and its associations with sociodemographic and clinical characteristics, and diabetes dis-tress among adults with early-onset type 2 diabetes (20-45 years) | Quantitative (Cross-sectional) |
| Hu et al., 2020 | Diabetes distress in young adults with type 2 diabetes: A  cross-sectional survey in China | China | *n* 18-30 = 10 (10.20%)  *n* 31-45 (56) (57.14%) | Total sample  Female: 33.67%  Diabetes duration: 46.94% had diabetes for <5 years | To investigate the diabetes distress in young adults with type 2 diabetes and to analyse its influencing factors | Quantitative (Cross-sectional) |
| Nielsen et al., 2020 | To tell or not to tell: disclosure and self-management among adults with early-onset type 2 diabetes: A qualitative study | Denmark | *n* = 15 (100%) | Age range: 21-45 years  Median age: 38 years (IQR = 6)  Female: 66.67%  Range diabetes duration: 0.5-22 years  Median diabetes duration: 3 years (IQR = 3) | To explore the disclosure of type 2 diabetes and how disclosure affects diabetes self-management and the psychosocial adjustment to life with diabetes among adults with early-onset type 2 diabetes | Quantitative (Cross-sectional) |
| Gopalan et al., 2021 | Disclosure of new type 2 diabetes diagnoses to younger adults: A qualitative study | United States of America | *n* = 41 (100%) | Mean age: 38.4 years (SD = 5.8)  Male: 51%  Black: 29%  Latinx: 24%  Multiple or other self-reported ethnicity/race: 17% | To explore diagnosis disclosure experiences and initial reactions of younger adults with newly diagnosed type 2 diabetes | Quantitative (Cross-sectional) |
| Middleton et al., 2021 | An enhanced SMS text message–based support and reminder program for young adults with type 2 diabetes (TEXT2U): Randomized controlled trial | Australia | *n =* 40 (100%) | Intervention group  Mean age: 33 years (SD = 5.8)  Male: 48%  Ethnicity:  East/Southeast Asian: 38%  Subcontinental: 24%  European: 19%  Other: 19%  Control group  Mean age: 32.4 years (SD = 4.4)  Male: 53%  Ethnicity:  East/Southeast Asian: 16%  Subcontinental: 37%  European: 32%  Other: 16% | To investigate the effectiveness of an enhanced SMS text message–based support and reminder programme in improving clinic attendance, metabolic control, engagement in self-management, and psychological health in young-onset type 2 diabetes (18-40 years) | Quantitative (Randomised controlled trial) |
| Park et al., 2021 | Questionnaire-based survey of demographic and clinical characteristics, health behaviours, and mental health of young Korean adults with early-onset diabetes | South Korea | *n* = 165 (73.3%) | Total sample  Mean age: 33.1 years (SD = 6.0)  Male: 51.6%  Mean age at diagnosis: 29.2 years (SD = 6.8)  Mean diabetes duration: 3.9 years (SD = 4.4) | To assess the clinical and demographic characteristics, health behaviours, and mental health of Korean adults with early-onset diabetes | Qualitative (Cross-sectional) |
| Riaz et al., 2021 | Risk of depression among early-onset type 2 diabetes mellitus patients | Bangladesh | *n =* 372 (32.5%) | Sample onset of diabetes <40 years  Age:  <50 years: 79%  50–59 years: 17.4%  ≥60 years: 3.6%  Female: 82.3%  Diabetes duration:  <5 years: 22.4%  5-10 years: 30.1%  ≥15 years: 15.7% | To examine the relationship between time of diagnosis and risk of depression | Mixed-methods |
| Celik et al., 2022 | ‘No one ever asks about something that actually is relevant to my life’: A qualitative study of diabetes and diabetes care experiences of young women with type 2 diabetes during their reproductive years | United Kingdom and Turkey | *n =* 36 (100%) | *n* from the United Kingdom = 23  Age range: 20–45 years  Median age: 37 years  Diabetes duration range: 1-21 years  Median diabetes  Duration: 4.5 years  Asian: 36.11% | To understand the experiences, views and the health needs of young women with type 2 diabetes during their reproductive years | Qualitative (Cross-sectional) |
| de Wit et al., 2022 | ISPAD Clinical Practice Consensus Guidelines 2022: Psychological care of children, adolescents and young adults with diabetes | N/A | N/A | N/A | To review relevant literature, and to outline clinical practice consensus guidelines for psychological care of children, adolescents and young adults with type 2 diabetes | Clinical guidelines |
| Dibato et al., 2022 | Temporal trends in the prevalence and incidence of depression and the interplay of comorbidities in patients with young- and usual-onset type 2 diabetes from the USA and the UK | United States of America and United Kingdom | *n* 18-39 years United Kingdom = 18,809  *n* 18-39 years United States of America = 84,851 | 18-39 years group  United Kingdom  Mean age: 33 years (SD = 5)  Men: 50%  United States of America  Mean age: 33 years (SD = 5)  Men: 33% | To investigate the prevalence and incidence of depression, and the interplay of cardiometabolic  comorbidities, in the risk of developing depression between young-onset diabetes (diagnosis at age <40 years) and usual onset diabetes (diagnosis at age ≥40 years) | Quantitative  (Retrospective cohort study) |
| Health Innovation Network Manchester, 2022 | Mapping the challenge of type 2 diabetes in Greater Manchester | United Kingdom | *n* (quant) = 43 (100%)  *n* (qual) = 17 (100%) | Not reported | To understand experiences and views of younger adults with type 2 diabetes living in Greater Manchester | Mixed-methods |
| Liu et al., 2022 | Early-onset type 2 diabetes and mood, anxiety, and stress-R  related disorders: A genetically informative register-based cohort study | Sweden | *n =* 7,896 (0.3%) | Median age diagnosis: 34.3 years (IQR = 28.4-39.1)  Male: 55.9% | To investigate the association  and familial coaggregation between early-onset type 2 diabetes and clinically diagnosed mood, anxiety, and stress-related disorders and to quantify contributions of genetic and environmental factors to these associations | Quantitative (Population-based cohort study) |
| Prothero et al. (2022) | Barriers and enablers to diabetic retinopathy screening: A cross-sectional survey of young adults with type 1 and type 2 diabetes in the UK | United Kingdom | *n =* 33 (33%) | Age:  18-23 years: 3%  24-29 years: 15.2%  30-34 years: 75.8%  Male: 51.5%  Diabetes duration:  <3 years: 33.3%  3-9 years: 57.6%  10-14: 9.1%  Ethnicity:  White British: 30.3%  Indian: 24.2%  Bangladeshi: 18.2%  Pakistani: 12.1%  Other: 12.1% | To identify barriers and enablers for diabetic retinopathy screening attendance among young adults living with type 1 and type 2 diabetes in the United Kingdom, and to explore differences between those that attend diabetic retinopathy screening regularly and those who do not | Quantitative (Cross-sectional) |
| Croke et al., 2023 | What are the perspectives of adults aged 18–40 living with type 2 diabetes in urban settings towards barriers and opportunities for better health and well-being: A mixed-methods study | United Kingdom | *n* = 46 (100%) | Mean age: 35.3 years (SD = 4.4)  Male: 67%  Ethnicity:  White: 74.4%  Asian/Asian British: 20.9%  Black/African/Caribbean/  Black British: 2.3%  Multiple ethnic groups: 2.3% | To understand the perspectives  and experiences of younger adults living with type 2 diabetes, to identify subgroups of adults who share specific perspectives towards health, well-being and living with diabetes, and to explore barriers to and opportunities for better diabetes prevention and management, and improved well-being | Mixed-methods |
| Barker et al., 2023 | Age at diagnosis of type 2 diabetes and depressive symptoms, diabetes-specific distress, and self-compassion | United Kingdom | *n =* 64 (9.1%) | Sample diagnosed <40 years  IQR age: 44–58 years  Mean age: 53 years  Women: 45.3%  IQR diabetes duration: 11-26 years  Median diabetes duration: 18 years  South Asian: 17.2% | To investigate the association between age at diagnosis and depression, diabetes-specific distress and self-compassion among adults with established type 2 diabetes | Quantitative (Cross-sectional) |
| Wong et al., 2023 | Diabetes knowledge, self-efficacy and dietary, psychological and physical health barriers: Comparing young and usual-onset type 2 diabetes | Singapore | *n* = 97 (23.72%) | Mean age: 38 years (SD = 5.5)  Mean diabetes duration: 4.5 years (SD = 3)  Male: 56.7%  Ethnicity:  Chinese: 49.5%  Malay: 29.9%  Indian: 17.5%  Other: 3.1% | To assess and compare knowledge, skills and barriers to self-management between young-onset type 2 diabetes and usual-onset diabetes | Qualitative (Cross-sectional) |
| Berry-Price, 2024 | Exploring the lived experience of Self-Care in young adults with type 2 diabetes | United States of America | *n =* 19 (100%) | Age:  18-24 years: 31.58%  25-30 years: 68.42%  Male: 68.42%  Ethnicity:  Black or African American: 84.21%  Latinx or Hispanic American: 5.26%  White or European American: 10.53% | To understand and describe self-care experiences of young adults with type 2 diabetes | Qualitative (Cross-sectional) |
| Cha et al., 2024 | Differences in patient-reported and clinical characteristics by  age group in adults with type 2 diabetes | South Korea | *n* = 27 (15.17%) | <50 group  Age range: 22-49 years  Mean age: 39.41 years (SD = 7.09)  Men: 55.56%  Mean diabetes duration: 7.7 years (SD = 5.25) | To explore potential differences in patient-reported and clinical characteristics by age group and to gain insights into the value of using an age-specific approach for diabetes self-management education | Quantitative (Cross-sectional) |
| Health Innovation Network South London, 2024 | Early-onset type 2 diabetes: Lived experience insights report | United Kingdom | *n* (quant) = 70 (100%)  *n* (qual) = 31 (100%) | Qual  Age:  26-34 years: 45%  35-39 years: 55%  Gender:  Female: 68%  Age at diagnosis:  ≤25 years: 32%  26-30 years: 24%  31-35 years: 44%  Ethnicity:  White: 32%  Asian/Asian British and mixed/multiple ethnic groups: 23%  Black/Black British, Caribbean or African: 45%  Quant  Age:  26-29 years: 10%  30-34 years: 34%  35-39 years: 56%  Gender:  Female: 67%  Age at diagnosis:  ≤25 years: 29%  26-30 years: 20%  31-35 years: 37%  36-40 years: 14%  Ethnicity:  White: 31%  Asian/Asian British: 19%  Black/Black British, Caribbean or African: 40%  Mixed/multiple ethnic groups and other ethnic group: 10% | To explore experiences of diabetes care in south east London of people living with type 2 diabetes aged between 26-39 years | Mixed-methods |
| Sim et al., 2024 | Understanding the preferences of young adults with type 2 diabetes mellitus with regard to diabetes self-management education: A qualitative study | Singapore | *n* = 21 (100%) | Age:  21-29 years: 23.8%  30-39 years: 76.2%  Male: 61.9%  Diabetes duration:  0-5 years: 71.4%  6-10 years: 28.6%  Ethnicity:  Chinese: 42.9%  Malay: 38.1%  Indian: 9.5%  Other: 9.5% | To understand the preferences of young adults with young-onset type 2 diabetes (21-39 years) in regards to the modality, content and qualities of diabetes self-management education | Qualitative (Cross-sectional) |
| Wong et al., 2024 | Identifying barriers and facilitators to self-care in young adults with type 2 diabetes | Singapore | *n* = 21 (100%) | Age range 23-39 years  Median age: 35 years  Women: 38.10%  Diagnosis duration:  1-2 years: 38.10%  3-5 years: 28.57%  >5 years: 33.33%  Ethnicity:  Chinese: 42.86%  Malay: 38.10%  Indian: 9.52%  Other: 9.52% | To map barriers and facilitators to self-care behaviours (healthy eating, regular exercise and medication adherence) in young-onset type 2 diabetes onto the domains of the Theoretical Domains Framework | Qualitative (Cross-sectional) |
| Javaherforooshzadeh et al., 2025 | Effectiveness of cognitive self-Compassion training on perceived stress and depression in patients with type 2 diabetes | Iran | *n* = 24 (100%) | Mean age: 34.91 years (SD = 4.61)  Male: 54.17% | To investigate the effectiveness of a self-compassion based cognitive intervention in reducing perceived stress and depression among adults (18-45 years) with type 2 diabetes | Quantitative (Quasi-experimental study) |
| Jang & Yang, 2025 | Effects of e-health literacy on health-related quality of life in young adults with type 2 diabetes: Parallel mediation of diabetes self-efficacy and  self-care behaviours | South Korea | *n* = 150 (100%) | Mean age: 33.21 years (SD = 4.43)  Women: 66.7%  Diabetes duration range: 6 months – >22 years  Mean diabetes duration: 3.84 years (SD = 3.56) | To investigate the impact of e-health literacy on health-related quality of life in young adults with type 2 diabetes, with a particular focus on the mediating roles of diabetes self-efficacy and self-care behaviours | Quantitative (Cross-sectional) |
| Ma et al., 2025 | The impact of diabetes stigma on psychological,  behavioral and clinical outcomes in young  and middle-aged Chinese patients with type  2 diabetes mellitus: The moderating effects of  psychosocial factors | China | Not reported | Total sample (Baseline)  Mean age: 43.96 years (SD = 11.08)  Male: 70%  Diabetes duration:  ≤ 2: 48%  3-5: 17%  6-10: 14%  >10: 21% | To investigate the associations between dia­betes stigma and psychological, behavioural, and clinical outcomes and the moderating effects of hope, self-esteem, and social sup­port in young and middle-aged Chinese adults with type 2 dia­betes mellitus | Quantitative (Cross-sectional) |
| Khavere et al., 2025 | Effectiveness of self-management interventions on Type  2 diabetes among young adults (18–45 years): A systematic review and meta-analysis | N/A | N/A | N/A | To investigate the effectiveness of type 2 diabetes self-management interventions among young adults (18-45 years) with type 2 diabetes | Systematic review and meta-analysis |
| Riise et al., 2025 | Diabetes distress and associated psychosocial factors in type 2 diabetes. A population-based  cross-sectional study. The HUNT study, Norway | Norway | *n* (<50 years) *=* 140 (7.2%) | Age group <50 years  Female: 57.9%  Diabetes duration:  <1 year: 3.6%  1-4 years:20.8%  5-9 years:19.9%  ≥10 years: 55.8% | To analyse the prevalence of diabetes distress among participants with type 2 diabetes, and to examine associations of demographic, lifestyle- and clinical  factors, including anxiety and depression, with diabetes distress, using data from the fourth wave of the population based Trøndelag Health (HUNT)  study in Norway | Quantitative (Cross-sectional) |
| Zhao et al., 2025 | Barriers and facilitators to physical activity in people  with young-onset (aged 18-40 years) type 2 diabetes: A qualitative study | China | *n* = 23 | Age range: 20-40 years  Median age: 29 years  Age IQR: 11 years (23-34)  Female: 56.5%  Diabetes duration range: 0.25-11 years  Median diabetes duration: 1 year  Diabetes duration IQR: 1.67 years (0.33-2) | To explore the experience and perceptions of physical  activity of people with young-onset type 2 diabetes, and to identify potential barriers and enablers to physical activity | Qualitative (Cross-sectional) |
| Chauhan et al., 2025 | Using normalisation process theory to understand implementation of effective early-onset type 2 diabetes treatment and care within England: A qualitative study | United Kingdom | *n* = 25 (50%) | Gender  Female: 76%  Age  16-24 years: 4%  25-39 years: 88%  40 years: 8%  Ethnicity  Asian or Asian British: 36%  White: 64%  Diabetes duration  <6 months: 12%  1-3 years: 24%  3-10 years: 60%  >10 years: 4% | To explore and to understand the unmet care needs of people living with early-onset type 2 diabetes and their diabetes | Qualitative study (Cross-sectional) |
| Goldney et al., 2025 | Designing a regional clinical service for people with early-onset type 2 diabetes in England | United Kingdom | N/A | N/A | To conduct a literature review to inform the design of a regional clinical service for people with early-onset type 2 diabetes in Leicester, Leicestershire and Rutland in England | Literature review |
| Strandberg et al., 2025 | Pharmacologically treated depression, anxiety, and insomnia in individuals with type 2 diabetes: The role of diabetes duration, age, and age at diabetes  onset. A Norwegian population-based registry study from the OMIT cohort | Norway | *n* = 6787 (12.32%) | Total sample  Mean age at diagnosis: 54.7 years (SD = 12.8)  Mean diabetes duration: 7.6 years (SD = 7.3)  Men: 58.08%  Immigrant background: 12.46% | To investigate the association between diabetes duration, use of medication for depression, anxiety, or insomnia and whether associations were influenced by age, age at diabetes onset, sex and education | Quantitative (Cross-sectional) |

*Note.* N/A = Not applicable; SD = Standard deviation.; IQR = Interquartile range.
