# Supplementary material for: Psychosocial care and experiences in young adults living with early‐onset type 2 diabetes: A narrative review
Source: Diabet Med. 2026 May 9;43(7):e70352. doi: 10.1111/dme.70352 (PMC13257897; doi:10.1111/dme.70352)
Supplement: Supplementary file 3 — Data S1. [file DME-43-e70352-s002.docx]

**Additional references (51-71 / S1-S21)**

S1. Armas A. *Primary care providers observations on diabetes management for young adults in Ontario Barriers and enablers to care and patient-provider communication*. University of Waterloo; 2017. Accessed 25/07/25. <https://uwspace.uwaterloo.ca/items/157c93f5-5a93-4995-92ba-21105f1a6ca1>

S2. Hagan J, Davies MJ, Speight J, Hadjiconstantinou M. Stigma associated with early-onset type 2 diabetes: A secondary qualitative analysis. *British journal of health psychology*. 2025;31(1)doi:10.1111/bjhp.70042

S3. Wong J, Ross GP, Zoungas S, et al. Management of type 2 diabetes in young adults aged 18-30 years: ADS/ADEA/APEG consensus statement. *The Medical Journal of Australia*. 2022;216(8):422-429. doi:10.5694/mja2.51482

S4. Berry-Price H. *Exploring the lived experience of self-care in young adults with type 2 diabetes*. East Tennessee State University; 2024. Accessed 18/07/2025. <https://dc.etsu.edu/etd/4347>

S5. Diabetes UK. Tackling Diabetes Stigma Position Statement: August 2025. Accessed March 02, 2026. <https://www.diabetes.org.uk/sites/default/files/2025-08/Tackling%20stigma%20-%20position%20paper%20Aug%202025%20-%20Copy.pdf>

S6. Ma T, Li A, Han Y, Jiang Q, Hou Y. The impact of diabetes stigma on psychological, behavioral and clinical outcomes in young and middle-aged Chinese patients with type 2 diabetes mellitus: the moderating effects of psychosocial factors. *Psychology & Health*. 2025;doi:10.1080/08870446.2025.2552234

S7. Savage S, Dabkowski S, Dunning T. The education and information needs of young adults with type 2 diabetes: A qualitative study. *Journal of Nursing and Healthcare of Chronic Illness*. 2009;1(4):321-330. doi:10.1111/j.1752-9824.2009.01035.x

S8. Arons A, Seligman H. The emerging adulthood gap in diabetes prevention research. *The Lancet Regional Health–Americas*. 2024;32

S9. Prothero L, Cartwright M, Lorencatto F, et al. Barriers and enablers to diabetic retinopathy screening: A cross-sectional survey of young adults with type 1 and type 2 diabetes in the UK. *BMJ Open Diabetes Research & Care*. 2022;10(6)doi:10.1136/bmjdrc-2022-002971

S10. Diabetes Australia. *Young adults with diabetes needs analysis*. 2006. Accessed March 02, 2026. <https://www.diabetesaustralia.com.au/wp-content/uploads/Young-Adults-with-Diabetes-Needs-Analysis.pdf>

S11. Wilmot EG, Idris I. Early onset type 2 diabetes: Risk factors, clinical impact and management. *Therapeutic Advances in Chronic Disease*. 2014;5(6)doi:10.1177/2040622314548679

S12. Liu S, Leone M, Ludvigsson JF, et al. Early-onset type 2 diabetes and mood, anxiety, and stress-related disorders: A genetically informative register-based cohort study. *Diabetes Care*. Dec 1 2022;45(12):2950-2956. doi:10.2337/dc22-1053

S13. Middleton T, Constantino M, McGill M, et al. An enhanced SMS text message-based support and reminder program for young adults with type 2 diabetes (TEXT2U): Randomized controlled trial. *Journal of Medical Internet Research*. 2021;23(10):e27263. doi:10.2196/27263

S14. Leicester Diabetes Centre. New early onset type 2 diabetes course supports treatment and care for young adults. Accessed March 02, 2026. <https://www.edendiabetes.com/news-blog/2024/10/21/new-early-onset-type-2-diabetes-nhs-online-course-for-healthcare-professionals-supports-treatment-and-care-for-young-adults>

S15. Baile WF, Buckman R, Lenzi R, Glober G, Estela BA, Andrzej KP. SPIKES-A six-step protocol for delivering bad news: application to the patient with cancer. *The oncologist*. 2000;5(4)doi:10.1634/theoncologist.5-4-302

S16. Wong SKW, Smith HE, Chua JJS, et al. Effectiveness of self-management interventions in young adults with type 1 and 2 diabetes: A systematic review and meta-analysis. *Diabetic Medicine*. Feb 2020;37(2):229-241. doi:10.1111/dme.14190

S17. Javaherforooshzadeh M, Ehteshamzadeh P, Hooman F, Bakhtiarpour S. Effectiveness of cognitive self-compassion training on perceived stress and depression in patients with type 2 diabetes. *Journal of Basic Research in Medical Sciences*. 2025;12(1):34-42. doi:10.61186/jbrms.12.1.34

S18. Khavere S, Hadjiconstantinou M, Miksza J, et al. Effectiveness of self-management interventions on Type 2 diabetes among young adults (18-45 years): A systematic review and meta-analysis. *Diabetic Medicine*. 2025:e70127. doi:10.1111/dme.70127

S19. Dibaiyan S, Sohrabi F, Poursharifi H, Sabet M. Comparison of the effectiveness between web-based motivational interviewing and web-based diabetes self-management education on glycemic control in diabetic patients type 2. *Razavi International Journal of Medicine*. 2022;10(4):31-36.

S20. Abdollahi S, Hatami M, Manesh FM, Askari P. The Effectiveness of Acceptance and Commitment Therapy on the Self-Care and Adherence to Treatment in Patients with Type 2 Diabetes. *International Archives of Health Sciences*. Apr-Jun 2020;7(2)doi:10.4103/iahs.iahs_13_20

S21. Diabetes UK. Addressing the care gap for young people with type 2 diabetes. Accessed March 02, 2026. <https://www.diabetes.org.uk/our-research/get-involved/take-part-in-research/care-young-people-type-2>
